# Can seafood from marine sites of dumped World War relicts be eaten?

**DOI:** 10.1007/s00204-021-03045-9

**Published:** 2021-04-10

**Authors:** Edmund Maser, Jennifer S. Strehse

**Affiliations:** Institute of Toxicology and Pharmacology for Natural Scientists, University Medical School Schleswig-Holstein, Campus Kiel, Brunswiker Str. 10, 24105 Kiel, Germany

**Keywords:** Dumped munitions, Marine environment, Biomonitoring, Blue mussels, Marine food chain, Risk assessment

## Abstract

Since World War I, considerable amounts of warfare materials have been dumped at seas worldwide. After more than 70 years of resting on the seabed, reports suggest that the metal shells of these munitions are corroding, such that explosive chemicals leak out and distribute in the marine environment. Explosives such as TNT (2,4,6-trinitrotoluene) and its derivatives are known for their toxicity and carcinogenicity, thereby posing a threat to the marine environment. Toxicity studies suggest that chemical components of munitions are unlikely to cause acute toxicity to marine organisms. However, there is increasing evidence that they can have sublethal and chronic effects in aquatic biota, especially in organisms that live directly on the sea floor or in subsurface substrates. Moreover, munition-dumping sites could serve as nursery habitats for young biota species, demanding special emphasis on all kinds of developing juvenile marine animals. Unfortunately, these chemicals may also enter the marine food chain and directly affect human health upon consuming contaminated seafood. While uptake and accumulation of toxic munition compounds in marine seafood species such as mussels and fish have already been shown, a reliable risk assessment for the human seafood consumer and the marine ecosphere is lacking and has not been performed until now. In this review, we compile the first data and landmarks for a reliable risk assessment for humans who consume seafood contaminated with munition compounds. We hereby follow the general guidelines for a toxicological risk assessment of food as suggested by authorities.

## Introduction

**Fig. 1:**
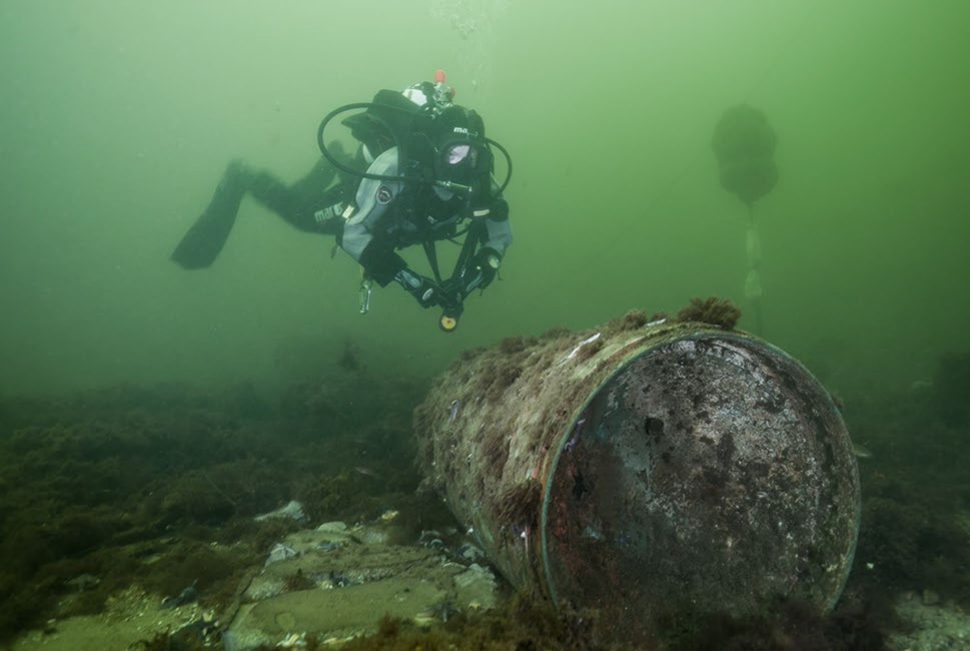
A scientific diver next to a submerged mine in the western Baltic Sea. © Jana Ulrich, FTZ CAU

**Fig. 2:**
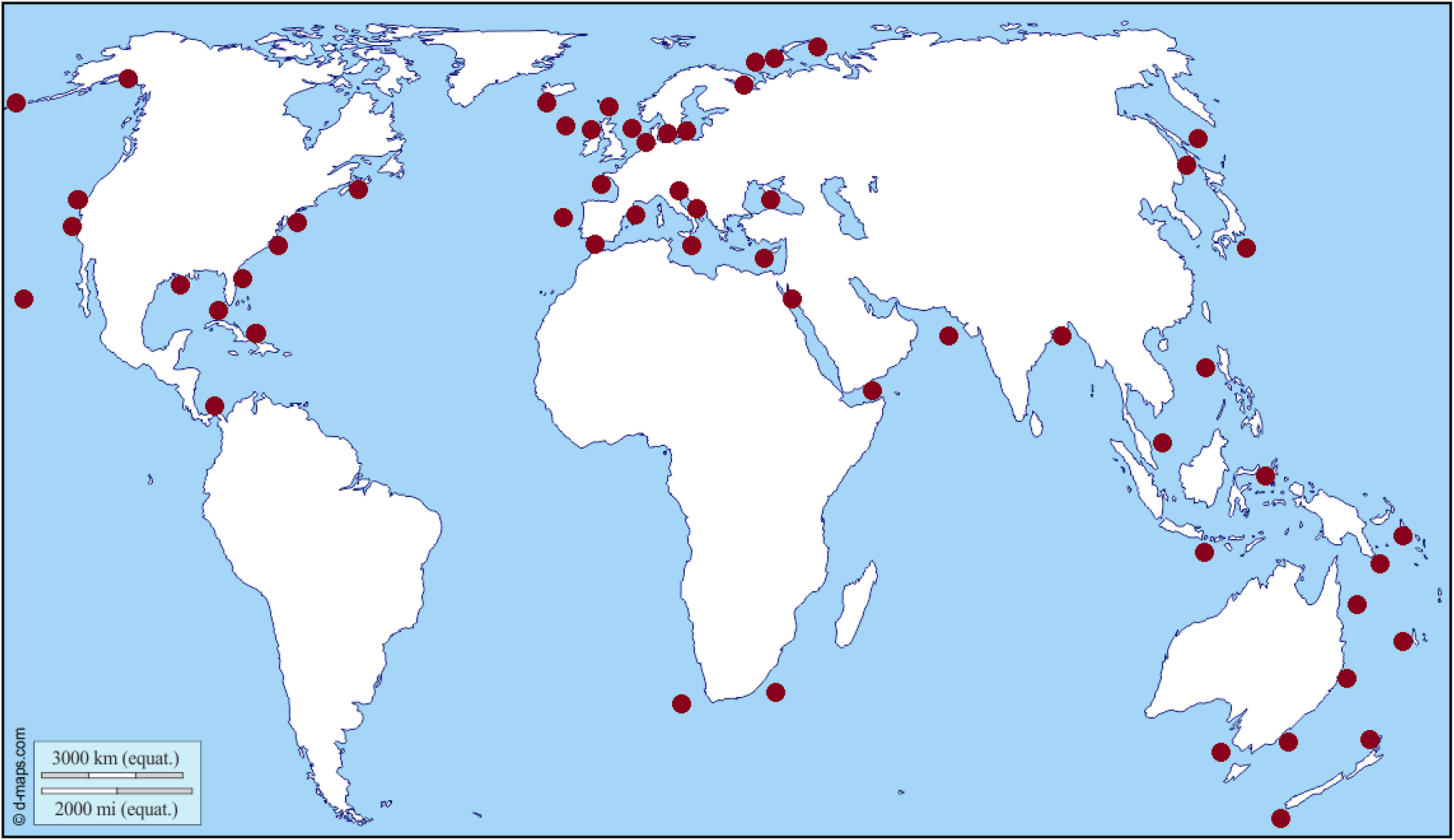
Examples for marine sites with munitions present (adapted from Beck et al. 2018) https://www.d-maps.com/carte.php?num_car=3266&lang=de date: July 2020

Seas worldwide are threatened by a newly identified source of pollution: millions of tons of all kinds of warfare materials which were dumped intentionally after World War I and II, in addition to mine barriers, failed detonations, as well as shot down military planes and ship wrecks carrying munitions (Beck et al. 2018). For example, in the German parts of the Northern and Baltic Sea alone nearly two million metric tons of toxic conventional explosives (TNT and others) and more than five thousand metric tons of chemical weapons are present (Fig. 1) (Böttcher et al. 2011). Munitions in the seas is also a worldwide problem, e.g., coastal areas on the Pacific Ocean, such as Australia and Asian countries and North and Middle American coastlines on the Pacific and Atlantic (Fig. 2) (MEDEA 1997; US Army 2001).

Increased human access, as observed in fisheries, wind farms and construction of pipelines, pose the risk of facilitating detonation of such unexploded ordnances. On the other hand, the metal shells of these munitions are corroding after more than 70 years of resting on the seabed, such that chemicals leak out and distribute in the marine environment. Explosive chemicals like TNT and its derivatives are known for their toxicity and carcinogenicity (Bolt et al. 2006; Koske et al. 2019).

Release of explosive chemicals and other munition-related compounds into the environment, resulting in contamination of surface and ground waters, soils and sediment, has been documented for several dumping sites throughout the world. (Talmage et al. 1999; Bełdowski et al. 2016; Edwards et al. 2016; Silva and Chock 2016; Jurczak and Fabisiak 2017). Due to their distribution in the oceans, munition compounds (MCs) are absorbed by aquatic organisms (Böttcher et al. 2011; Strehse et al. 2017; Appel et al. 2018; Beck et al. 2019; Maser and Strehse 2020; Schuster et al. 2020) and pose a threat to both the marine ecosphere and the human seafood consumer. Therefore beside identification, mapping and monitoring, environmental and human safety risk assessments, as outlined in this commentary, are urgently needed.

## Toxic effects on marine organisms

In both lab- and field-based studies, uptake and accumulation of toxic explosive chemicals in marine animals has been proven, while lab-based studies also showed that uptake of explosives positively correlated with their exposure concentrations. (Rosen and Lotufo 2007; Ek et al. 2008; Strehse et al. 2017; Maser and Strehse 2020; Schuster et al. 2020). TNT, (2,4,6-trinitrotoluene) as the “parent” compound leaching from corroding munitions or free lying chunks of hexanite, undergoes metabolic transformation processes via photolysis, hydrolysis, oxidation and reduction to yield its main metabolites 2-ADNT (2-amino-4,6-dinitrotoluene), 4-ADNT (4-amino-2,6-dinitrotoluene) and 2,4-DA-6-NT (2,4-diamino-6-nitrotoluene) (Goodfellow et al. 1983; Beck et al. 2018). It is currently debated in the scientific community whether TNT is transformed to its metabolites by UV light, microorganisms living in the seafloor sediment or on the surface of the biota, or by metabolic activities of detoxification enzymes in the target species (Beck et al. 2018; Strehse et al. 2020). Howsoever, there is some evidence that TNT metabolites 2-ADNT, 4-ADNT and 2,4-DA-6-NT may be more toxic than the parent compound. Nonetheless, reliable data on the extent of exposure of the marine environment to these compounds are limited or lacking (Lotufo et al. 2013).

So far, TNT and its metabolites have been found in the bile of the common flatfish dab (*Limanda limanda* L.) living in the dump site Kolberger Heide at the Baltic Sea (Koske et al. 2020). These samples revealed dramatically enhanced tumour rates, compared to samples obtained from a reference site (Straumer and Lang 2019). Likewise in cod caught from the Bornholm Deep, traces of chemical warfare agents were detected, but these were found in muscle tissue, in contrast to the explosive compounds found in dab bile (Niemikoski et al. 2020).

Also, mussels take up MCs and, due to their nature as sedentary and filter-feeding organisms, have recently been developed as a useful biomonitoring model to determine explosive chemical contaminations leaking from corroding munitions (Strehse et al. 2017; Maser and Strehse 2020). Moreover, the blue mussel is an important seafood species and can thus simultaneously be used as an indicator to assess the entry of toxic substances into the marine food chain (Farrington et al. 1983). Unexpectedly, mussels caged in the immediate vicinity of lumps of TNT showed tissue concentrations of TNT metabolites, thus excluding them from human consumption because of toxicological concerns (Strehse et al. 2017; Maser and Strehse 2020) (See below). These results unequivocally show that the proximity of dumped munitions to marine biota influences entry of explosive chemicals into such organisms (Strehse et al. 2017; Appel et al. 2018; Maser and Strehse 2020).

Toxicity studies suggest that MCs are unlikely to cause acute toxicity to marine organisms at munition-contaminated sites due to their slow dissolution and high dilution (Beck et al. 2018, 2019). However, there is increasing evidence that munition chemicals can have sublethal and chronic effects in aquatic biota, especially in organisms that live directly on the sea floor or in subsurface substrates (Talmage et al. 1999; Juhasz and Naidu 2007; Lotufo et al. 2013). Several laboratory experiments have shown the toxicity of TNT in aquatic organisms (Talmage et al. 1999; Ek et al. 2005; Juhasz and Naidu 2007; Rosen and Lotufo 2007; Koske et al. 2019). Sublethal responses to TNT exposure generally include reduced growth and reproduction, impaired development, and damage to the nervous, immune and blood systems (Gong et al. 2007). More specifically, impacts on the zoospore germination, germling length and cell number have been observed in the green macroalga *Ulva fasciata*, survival and reproductive success in the polychaete *Dinophilus gyrociliatus*, embryo development and byssal threat form in the mollusc *Mytilus galloprovincialis* as well as survival of the opossum shrimp *Americamysis bahia* and the redfish *Sciaenops ocellatus* (Nipper et al. 2001; Rosen and Lotufo 2007).

## Toxic and carcinogenic effects on humans

Uptake of explosive chemicals by organisms, following exposure to underwater munitions, may cause their entry into the marine food chain and directly affect human health. The mechanism by which TNT and its metabolites exert toxic effects on a large number of organs in humans has not been fully elucidated. With chronic occupational exposure, typical effects were methemoglobin formation up to cyanosis, anemia, damage to bone marrow and spleen, cataract formation (TNT star), dermatitis, hepatitis and toxic polyneuritis (Ryon and Ross 1990). Damage to the hematopoietic system and the liver was also found in animals (Bolt et al. 2006; Naumenko et al. 2017). TNT has been tested for carcinogenicity in 2-year bioassays in rats and mice. After administration of TNT via diet, carcinoma of the urinary bladder and hepatocellular neoplasms were observed in rats, while malignant lymphoma combined with lymphocytic and granulocytic leukemia in the spleen significantly increased in mice. US-EPA concluded that TNT is a possible human carcinogen (Class C) (Bolt et al. 2006). A study in humans found elevated levels of chromosomal aberrations in a subset of TNT-exposed workers who were also positive for *N*-acetyltransferase (*NAT1*) (rapid acetylator) and exhibited the null glutathione-*S*-transferase (GST) T1 (*GSTT1*) or *GSTM1* genotype (Sabbioni and Rumler 2007). In Germany, TNT has been classified as belonging to MAK Group 2 (“substances that are considered to be carcinogenic in humans”).

## Toxicological risk assessment for munitions in seas

Leaking and bioaccumulation of toxic chemicals from corrosive munitions pose a threat to the marine ecosystem. In addition, these chemicals may enter the marine food chain and directly affect human health. Therefore, a toxicological risk assessment is of relevance to the marine ecosphere and the human seafood consumer. The assessment requires two different sets of data. Firstly, the hazard potential of a given chemical has to be determined by experimental investigations to define the toxicological endpoint in specific target tissues in animal and human studies (liver, kidney, blood, brain, eyes, skin). Secondly, the exposure has to be estimated, i.e., the nature and extent to which animals or human individuals are exposed to chemicals. From the combined assessment of the hazard potential and exposure, the actual risk is derived.

## Eco-toxicological risk assessment of MCs in marine organisms

To calculate the eco-toxicological risk, a combination of four approaches is used and these are: (1) the results of laboratory experiments using various biota species (fish, mussels, crustaceans, algae) from aquarium studies; (2) literature values of EC_50_ (50% of animals show the anticipated effect) and LD_50_ (50% of animals die) in the same species; (3) concentrations of MCs measured in various biota species found or exposed in field studies, together with results of water and sediment analyses; and (4) data on temporal variation through seasons, currents and extreme events such as storms in the dumping areas, but also consideration for future climate change scenarios. Likewise, effect monitoring results (impaired growth and development, genetic aberrations, etc.) and molecular biomarker data (oxidative stress, etc.) recently reported (Strehse et al. 2017) should be considered.

**Fig. 3:**
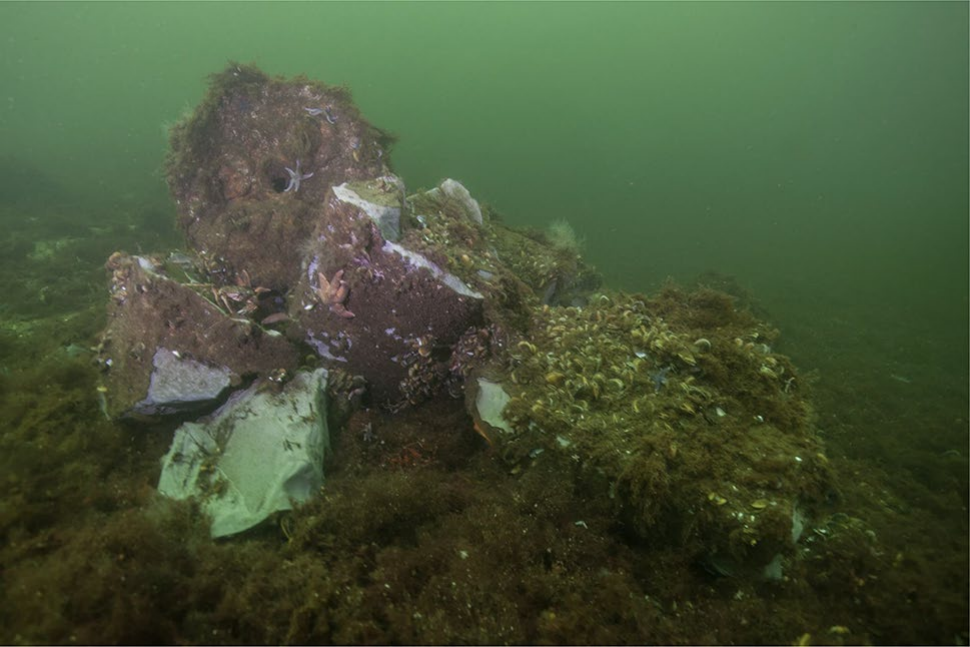
Chunks of hexanite (German Schiesswolle) with starfish, mussels and other kind of marine fouling on the surface © Jana Ulrich, FTZ CAU

An interesting aspect is that all larger and smaller objects in aquatic systems are immediately populated as a habitat and/or used as a breeding ground for offsprings. This can lead to very high exposure scenarios, especially at locations where high concentrations of environmental pollutants are present. Beck et al. (2019) measured concentrations of 3 mg TNT per liter sea water directly on large chunks of hexanite (German “Schiesswolle” consisting of 45–67% TNT, 5–24% hexanitrodiphenylamine and 16–25% aluminium powder) in a munitions-dumping site at Kolberger Heide in the Kiel Bight (German part of the Baltic Sea). Although these high concentrations were more than a thousand times dilute in the vicinity of the dumping sites (just a few centimetres away), they could have a lethal effect on the development of fish eggs, fish larvae and young fish, since fish live in these areas and may choose to spawn in the caves and crevices between chunks of hexanite (Fig. 3). From laboratory aquarium experiments, it is known that the LD_50_ value of TNT for fish (the dose at which 50 percent of fish die) fluctuates between 0.8 and 3.7 mg TNT/L (Talmage et al. 1999). Especially in deep but calm water, TNT concentrations of this magnitude can occur in the immediate vicinity of sunken and damaged munitions (EK et al. 2008).

As a first conclusion with regard to the toxic effects on marine organisms, further research, especially in organisms that live directly on the sea floor or in subsurface substrates, is needed for a more precise risk assessment of MCs in the marine ecosphere. It should also be considered that munitions dumping sites could serve as nursery habitat for young biota species, demanding special emphasis on developing juvenile marine animals.

## A risk assessment for the human seafood consumer

Generally for non-carcinogenic effects, the NOAEL (“no observed adverse effect level”—which indicates the dose at which no toxic effect occurs) and LOAEL (“lowest observed adverse effect level”—which indicates the lowest dose with observed toxic effect) are given in data bases (e.g., BfR, EU, EFSA). For example, the US EPA set a Reference Dose for TNT at 0.5 μg/kg b.w. × day based on a study in which dogs were exposed to TNT for 26 weeks, with hepatotoxic effects considered as critical effects (US-EPA 1988).

However, TNT and its metabolites are even known to be carcinogenic; with TNT recently categorised as Group 2 by the “German MAK Commission” (MAK 2019). Because of the carcinogenicity of TNT and its metabolites with non-threshold effects, health risk assessment has to be performed using the margin of exposure (MOE) concept (EPA IRIS 1993; EPA 2012). The MOE indicates the ratio of the smallest effect dose in animal experiments to the level of human exposure. For safety, a BMDL10 (“Benchmark Dose Lower Confidence Limit 10%” which indicates a 10% higher incidence of cancer in a given tissue compared to control animals)-related MOE should normally be greater than 10,000 (EPA 2012).

Therefore in terms of seafood, the major question is whether the consumption of contaminated seafood from munitions dumpsites is safe for human consumers. For risk assessments, the hazard potential and toxicological end points of explosive chemicals will be derived from the literature. These literature data are calculated against the concentrations of explosive chemicals measured in relevant seafood species in field studies at dumping sites. Finally, the risk for the human seafood consumer is calculated considering the average seafood consumption of the human population (95th percentile of average). Since only two reliable studies on the carcinogenicity of TNT in animal studies are available (Furedi et al. 1984a, b), the T25 method will be applied to define the point of departure (POD) and to infer a possible health risk for the human seafood consumer instead of the BMDL10 concept (ECETOC 2002). More specifically, for calculating the carcinogenic risk, the following parameters will be used: (1) the concentration of TNT and its metabolites 2-ADNT, 4-ADNT and 2,4-DA-6-NT measured in the seafood species; (2) the per capita consumption of fish or seafood at 39 g per day in Germany (FIZ 2017), and (3) the carcinogenicity of TNT determined from animal experiments (rats: 50 mg/kg b.w. per day; mouse: 1.5 mg/kg b.w. per day) (Furedi et al. 1984a, b). Since there are only limited or no data available on the carcinogenicity of 2-ADNT, 4-ADNT and 2,4-DA-6-NT, the risk related to dietary exposure to these compounds will be assessed equal to the carcinogenicity for TNT and calculated as the sum of all these compounds. Details on the health risks associated with the toxicity and carcinogenicity of explosives such as RDX, HMX and metabolites thereof should be assessed accordingly. All data on the body burden of MCs in various biota species that will be collected in laboratory studies or field trials will be used to map possible bioaccumulation along the marine food chain, with humans as the top predator in this case. For this purpose, the contents of the individual species or individual organs and/or tissues of larger species could be evaluated separately and assessed along the trophic order (plankton [algae] < crustaceans [small to large] < mussels < fish [small to large] < top predators [porpoises, seals, birds, humans].

In a recent publication, Maser and Strehse (2020) carried out preliminary risk assessment for the consumption of Baltic Sea mussels from the Kolberger Heide dumping area (Kiel Bight). Overall, this preliminary toxicological risk assessment of the mussels resulted in two different scenarios: (1) the regular consumption of mussels that were exposed in the immediate vicinity of the corrosive mines (containing up to 5 ng explosives per g of tissue w.w.) (Appel et al. 2018) may not lead to an increase in the risk of cancer for humans, since the calculated MOE was higher than 25,000. In contrast, (2) mussels that were exposed in the immediate vicinity of smaller and larger lumps of exposed explosives contained significantly higher amounts of MCs (up to 350 ng/g of tissue w.w.) (Strehse et al. 2017; Maser and Strehse 2020). In the second scenario, the calculated MOEs were below 25,000, which would mean an increased risk of cancer with regular consumption of these mussels. It must be clearly emphasized here, however, that these preliminary calculations were carried out on the basis of a “worst-case” scenario where all explosive-related chemicals found in the mussels (2-ADNT, 4-ADNT, DANT) were considered to be as carcinogenic as TNT. Secondly, it was assumed that the average daily intake of fish and seafood of 39 g per person in Germany (FIZ 2017) consists only of these highly contaminated mussels. And thirdly, it was believed that affected people would consume these mussels on a daily basis throughout their lives (an average of 70 years) (Maser and Strehse 2020). In other words since the lifelong daily consumption of 39 g of mussels from the immediate vicinity of chunks of explosives lying freely on the sea bed is very unlikely, the consumption of mussels from the Baltic Sea can be described as safe from today's point of view.

However, since time-dependent assessments that could be used to estimate the risks of mussel consumption in the years ahead are currently unavailable, this statement may not be valid in the future in a worst case scenario. Moreover, when considering the corrosion rates from the past to foresee a rising risk for tomorrow requires a biomonitoring system that allows continuous observation of critical munition-dumping sites, and this system could serve as an early warning system for the human seafood consumer. This could ideally be performed with mussels, because mussels are filter-feeding organisms, are easy to handle and, in addition, are an important sea-food species (Strehse and Maser 2020).

While, e.g., a preliminary risk assessment for human consumption of mussels has been carried out (Maser and Strehse 2020), this is currently not (yet) possible for fish. TNT and metabolites as well as RDX and HMX were found in the bile of flatfish from the Kolberger Heide dumping area (Koske et al. 2020), but fish bile is usually not consumed by humans. The corresponding values in fish fillet are urgently needed for human risk assessment. However, it could be shown that these affected fish had an increased tumor rate in the livers (see above) (Straumer and Lang 2019).

Again, only by establishing a reliable monitoring system can predications be made regarding the safety of seafood consumption in humans. Until then, the precautionary principle should be deployed and aquaculture systems or fishing activities should strictly be avoided or even forbidden in the vicinity of munition-dumping sites.

## Conclusions

As the seas worldwide are impacted by underwater munitions either dumped in the seas or deployed during combat, these pose an environmental health risk and impair the sustainable economic development of all coastal waters. MCs affect the environment directly with regard to biodiversity and the marine food web, and indirectly affect socio-economic issues by impairing commercial fishing through contaminated seafood.

After all, there is an undisputable direct link between the occurrence of dumped munitions and increased concentrations of toxic substances with implications for the edibility of fish, mussels and other seafood. For a reliable risk assessment, further key species representing different levels of the marine food chain should be investigated and used to model the marine food web, and anticipate possible risks to the human seafood consumer. This requires both field- and lab-based studies, including the development of molecular biomarkers as early warning systems for biomonitoring strategies.

Therefore, concerted efforts are required to address the worldwide problem of underwater munitions and their entry into the marine food chain. These efforts should comprise measures, such as characterization, quantification and digital mapping of underwater munitions sites; assessment of their toxicological risk for both the marine ecosphere and the human seafood consumer; development of modeling and predictive tools to evaluate current and future risks, including scenarios with respect to global warming and, finally, the development of environmentally sound remediation methods without endangering human life.
